# Topical sirolimus solution for lingual microcystic lymphatic malformations in children and adults (TOPGUN): study protocol for a multicenter, randomized, assessor-blinded, controlled, stepped-wedge clinical trial

**DOI:** 10.1186/s13063-022-06365-y

**Published:** 2022-07-08

**Authors:** A. Marchand, A. Caille, V. Gissot, B. Giraudeau, C. Lengelle, H. Bourgoin, B. Largeau, S. Leducq, A. Maruani

**Affiliations:** 1 Department of Dermatology and Reference Center for Rare Diseases and Vascular Malformations (MAGEC), CHRU Tours, Avenue de la République, 37044 Tours, Cedex 9 France; 2 Clinical Investigation Center, INSERM 1415, CHRU Tours, 37000 Tours, France; 3 INSERM U1246 -SPHERE « MethodS in Patients-centered outcomes and HEalth REsearch », University of Nantes, University of Tours, 37000 Tours, France; 4 Pharmacovigilance Regional Centre (CRPV), CHRU Tours, 37000 Tours, France; 5 Department of Pharmacy, University Hospital Center of Tours, 37000 Tours, France

**Keywords:** Lingual microcystic lymphatic malformation, Lymphangioma, Lymphatic abnormalities, Sirolimus, Mucosa, Topical treatment, Stepped-wedge design

## Abstract

**Background:**

Lingual microcystic lymphatic malformations (LMLMs) are rare congenital vascular malformations presenting as clusters of cysts filled with lymph fluid or blood. Even small well-limited lesions can be responsible for a heavy burden, inducing pain, aesthetic prejudice, or oozing, bleeding, infections. The natural history of LMLMs is progressive worsening punctuated by acute flares. Therapeutic options include surgery, laser excision, and radiofrequency ablation but all are potentially detrimental and expose to local relapse. Therefore, the management frequently relies on a “watchful waiting” approach. In complicated LMLMs, treatment with oral sirolimus, a mammalian target of rapamycin (mTOR) inhibitor, is often used. Topical applications of sirolimus on the buccal mucosae have been reported in other oral diseases with good tolerance and none to slight detectable blood sirolimus concentrations. We aim to evaluate the efficacy and safety of a 1 mg/mL sirolimus solution applied once daily on LMLM of any stage in children and adults after 4, 8, 12, 16, 20, and 24 weeks of treatment compared to usual care (no treatment).

**Methods:**

This is a randomized, multicentric study using an individually randomized stepped-wedge design over 24 weeks to evaluate topical application of a 1 mg/mL sirolimus solution once daily, on LMLM, versus usual care (no treatment), the control condition. Participants begin with an observational period and later switch to the intervention at a randomized time (week 0, 4, 8, or 12). Visits occur every 4 weeks, either in the study center or by teleconsulting. The primary outcome will be the evaluation of global severity of the LMLM on monthly standardized photographs by 3 independent blinded experts using the physical global assessment (PGA) 0 to 5 scale. Secondary outcomes will include lesion size measurement and quality of life assessment, investigator, and patient-assessed global disease and specific symptoms (oozing, bleeding, sialorrhea, eating impairment, taste modification, aesthetic impairment, pain, and global discomfort) assessment. A biological monitoring will be performed including residual blood sirolimus concentration and usual laboratory parameters.

**Discussion:**

Given the disappointing state of current treatment options in LMLMs, topical sirolimus could become firstline therapy in treating LMLMs if its efficacy and safety were to be demonstrated.

**Trial registration:**

ClinicalTrials.gov NCT04128722. Registered on 24 September 2019.

EudraCT: EUCTR2019-001530-33-FR

Sponsor (University Hospital Center of Tours – CHRU Tours): DR190041-TOPGUN

French regulatory authorities: ID RCB: 2019-001530-33

**Supplementary Information:**

The online version contains supplementary material available at 10.1186/s13063-022-06365-y.

## Background

Lymphatic malformations (LMs) are rare congenital anomalies (estimated prevalence < 0.1%) [1]. They belong to the wider group of vascular malformations, according to the International Society for the Study of Vascular Anomalies (ISSVA) 2014 classification [2, 3], as part of low-flow vascular malformations. They can be isolated or associated with other anomalies or birth defects such as Proteus syndrome or CLAPO syndrome. LMs present as clusters of cysts filled with translucid fluid or blood that can be macrocystic, microcystic, or mixed type. Any part of the skin or mucosae can be involved, with or without involvement of the underlying deep structures. At least 50% of LMs can be found in the head and neck area [4]. In this area, the buccal cavity is the most often involved, especially in medial microcystic LMs of the tongue [5, 6]. Despite their very heterogenous clinical severity and presentations, all medial microcystic LMs are classified as stage III according to de Serres et al., which implies high post-surgical relapse rate and peri-operative complications [7].

Specifically, patients with LMLMs, even those with small well-limited lesions, often experience a heavy burden because of oozing, spontaneous bleeding, infections, or even speech, chewing, or breathing impairment. An important aesthetic prejudice is also frequently reported [8, 9]. The disease’s natural history features progressive worsening, often punctuated by acute flares such as infections, bleeding, or acute swelling, sometimes secondary to lymphatic drainage-stimulating conditions such as trauma or ear-nose-throat regional infections [9].

Current therapeutic options are still limited and mostly rely on surgery or local interventional procedures. Although sclerotherapy may yield good results in macrocystic LMs, where large well-delimited cysts, this standard treatment option remains disappointing in more ill-defined infiltrative microcystic LMs, with high rates of post-surgical relapse or deep-structure iatrogenic lesions [10–12].

Complete surgical resection is rarely achievable in the oral cavity and can be tied to significant post-operative complications, such as post-operative edema, post-operative infections, delayed healing, or facial nerve palsies in deep infiltrative oral LMs [10]. Multiple partial resections may achieve functional improvement while minimizing drawbacks.

Other interventional techniques reported include CO_2_-laser excision or radiofrequency ablation, most often for superficial LMs or as combination therapy after surgery for larger LMs. However, they often lead to reduced symptoms but only very rarely achieve permanent curative results and may induce deeper tissue scarring that might hamper secondary function-preserving surgery [6].

Thus, LMLM management remains challenging, and a conservative management (“wait-and-see” approach) is frequently chosen. Iterative courses of antibiotics and steroids are started in response to acute flares or infections, with the caveat that infection-induced LMLM growth may render secondary ablation difficult.

The mammalian target of rapamycin (mTOR) is an intracellular kinase downstream from the PI3K-AKT pathway. When activated by the effect of the PI3K-AKT complex, it binds with other intracellular proteins, forming serine-threonine kinase mTOR complexes 1 and 2, which are involved in cell growth and apoptosis via protein synthesis and cell-cycle regulation [13, 14]. The PI3K-AKT-mTOR pathway is heavily involved during angiogenesis and lymphangiogenesis [15].

Known mutations in the PI3K-AKT-mTOR pathway have been discovered in both non-syndromic and syndromic lymphatic abnormalities, such as Proteus syndrome and Pi3K-related overgrowth spectrum-linked diseases (CLOVES syndrome or Klippel-Trenaunay syndrome) [16–18].

Sirolimus is a powerful inhibitor of the mTOR kinase, thereby preventing cellular growth and leading to apoptosis. It has been granted US Food and Drug Administration approval for preventing chronic allograft rejection in renal transplant recipient. In vitro, applying sirolimus to rat pancreas cancer-derived cells reduced VEGF-C expression and inhibited lymphangiogenesis; similar results were found in mice inoculated with the cells [15]. Similarly, sirolimus-treated cells from LMs showed inhibited growth [17], whether cultivated from syndromic (Proteus syndrome) or isolated LMs.

The first oral sirolimus administration as a compassionate treatment in refractory LMs was reported in 2008 in a 9-month-old boy [19] with multiorgan failure due to Proteus syndrome. Since then, sirolimus has been applied in different vascular anomalies [9, 20, 21], with 2 RCTs of LMs currently under way (NCT00975819, Adams, USA [active] and NCT02509468, Maruani, France [completed]) [22, 23].

However, despite an overall good safety profile (absent or mild side effects in 2 of 3 patients with vascular anomalies, including children), significant dermatologic (oral mucositis), hepatic (elevated liver enzymes, hepatitis), or hematologic side effects may occur. Grade III or IV drug-induced cytopenia is reported in up to 27% of treated patients, with some severe infectious complications also being reported [9, 20].

Hence, close clinical and biological follow-up is required, with sirolimus blood concentration monitoring. Treatment indications tend to be limited to the most severe lesions (i.e., extensive deep infiltration, aerodigestive airway involvement).

### Topical sirolimus on skin and mucosae

The first topical use of sirolimus was reported in a 2005 pilot study involving 24 patients with plaque psoriasis [24]. It has also been tried in multiple inflammatory or vascular-based skin and mucosae diseases such as oral erosive lichen planus [25], oral pemphigus vulgaris [26], port-wine stains, or tuberous sclerosis-induced facial angiofibromas [27–29].

There are few case reports describing sirolimus use in cutaneous microcystic LMs, in both children and adults, yielding promising results regarding lesion shrinking and a sharp reduction in the frequency of acute bleeding, swelling, or infectious episodes [30–32].

Mucosal topical sirolimus treatment has been reported in some inflammatory conditions. Undiluted commercial oral sirolimus solution (Rapamune®, Pfizer) had moderate effect in seven females with oral and/or genital lichen planus [25]. The tolerance was overall good, with only one treatment interruption due to local irritation. Blood sirolimus levels were monitored, with a peak sirolimus concentration of 1.5 ng/mL, well below usual therapeutic concentrations of 4 to 12 ng/mL, in only one patient with both oral and genital involvement, 2 h after treatment.

Nudelmann et al. [33] performed more extensive studies of oral sirolimus as a 0.05% mouthwash. Therapeutic sirolimus concentrations were detected in saliva up to 4 h after mouth washing, and peak blood sirolimus concentrations were from 0.2 to 1 ng/mL at 1 h after a single rinse with sirolimus mouthwash. No immediate side effects were reported, and no significant change was reported between an oral cavity examination performed by an oral medicine specialist before and 24 h after using the mouthwash.

### Objectives

Hence, we aim to perform a clinical trial (TOPical sirolimus in linGUal microcystic lymphatic malformatioN [TOPGUN]) to assess the efficacy and safety of a 1-mg/mL sirolimus solution applied once daily as a topical treatment for oral microcystic LMs with lingual involvement in children and adults compared to usual care (no treatment).

## Methods

### Trial design

TOPGUN is a randomized, open, multicenter study with an individually randomized stepped-wedge design [34] over a 24-week period to evaluate the following:

- Daily topical application of 1 mg/mL sirolimus solution 0.5 to 1 mL according to the size of the LMLM lesion, the experimental intervention, *versus*
- Usual care (no treatment), the control condition

In this design, participants are included in a cohort in which treatment is introduced at a randomized timepoint. Included participants start the intervention in a staggered fashion over time. Randomization is balanced, so each participant has the same probability of starting the intervention at a given step as any other participant over the entire study period. Four steps are planned for starting the intervention (i.e., week 0 [W0], W4, W8 and W12). Hence, 3 participants will be randomly assigned to begin the intervention on W0, 3 on W4, 3 on W8, and 3 on W12.

In order to minimize participant burden related to the follow-up visits and procedures, we tried to reduce the number of on-site visits thanks to phone calls, especially during the control period (see Tables 1, 2, 3 and 4). Similarly, blood tests may be performed in partner local laboratories, closer to patients’ homes, which will then provide results to the investigator.

**Table 1:**
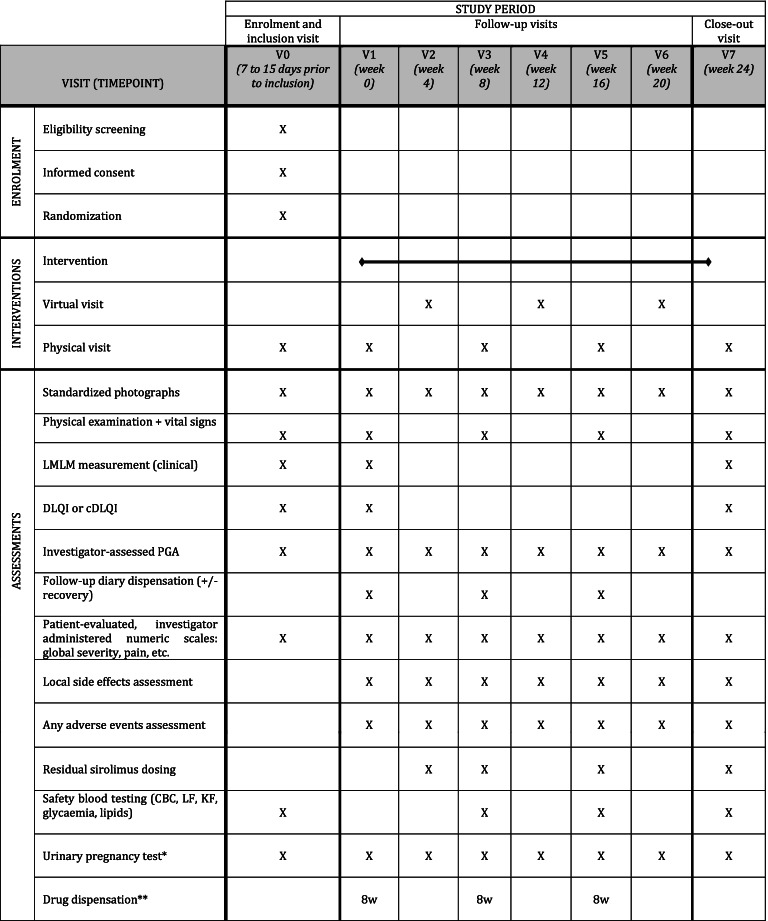
Participant timeline (group 1: treatment introduction at week 0)

^a^ For women of childbearing potential, until 3 months after study close-up

^b^ Drug dispensation: 3 bottles for 8 weeks (8w) or 2 bottles for 4 weeks (4w)

**Table 2:**
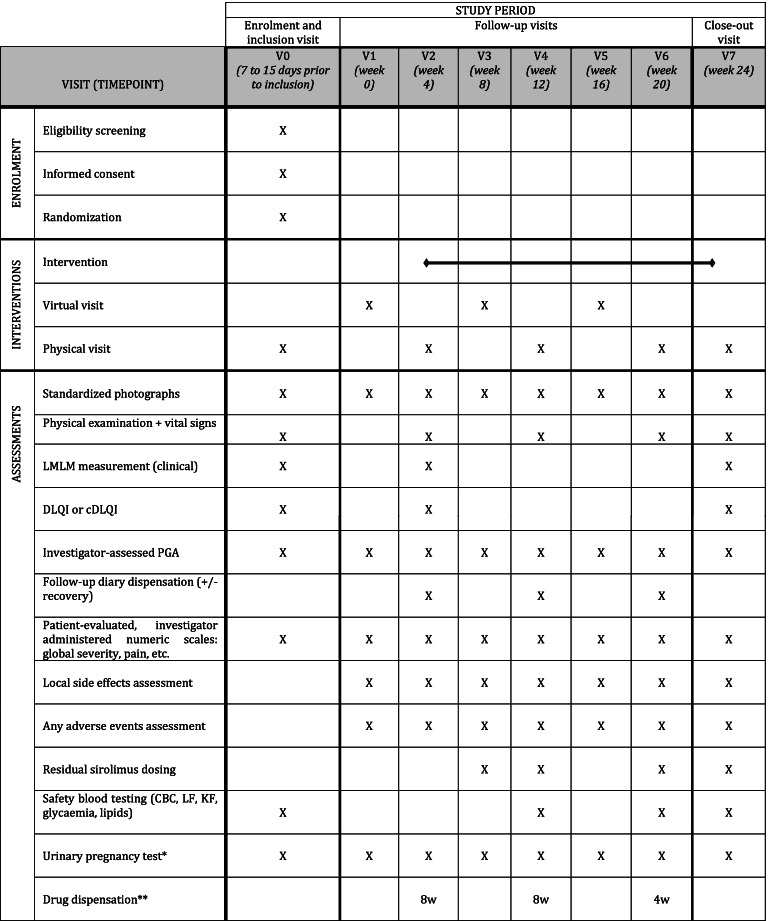
Participant Timeline (group 2: treatment introduction at week 4)

^a^ For women of childbearing potential, until 3 months after study close-up

^b^ Drug dispensation: 3 bottles for 8 weeks (8w) or 2 bottles for 4 weeks (4w)

**Table 3:**
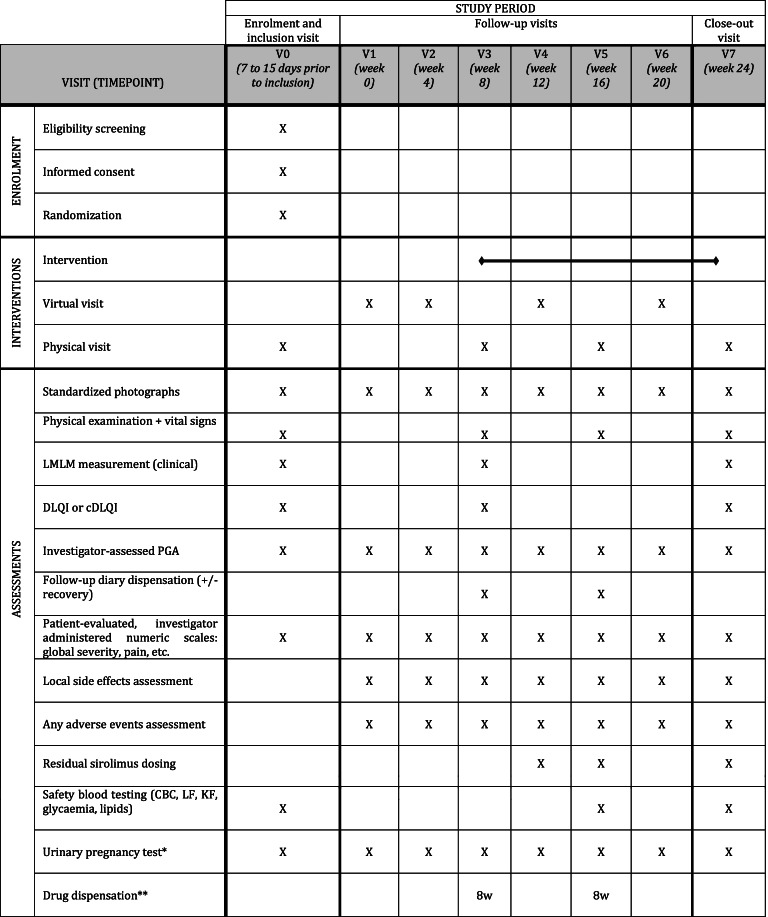
Participant Timeline (group 3: treatment introduction at week 8)

^a^ For women of childbearing potential, until 3 months after study close-up

^b^ Drug dispensation: 3 bottles for 8 weeks (8w) or 2 bottles for 4 weeks (4w)

**Table 4:**
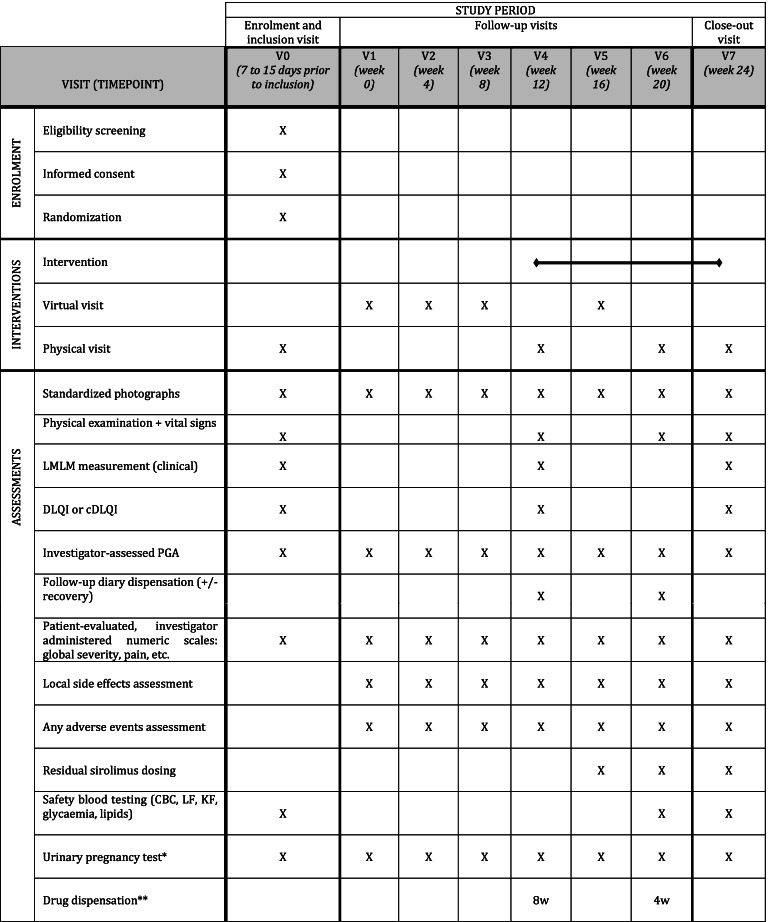
Participant Timeline (group 4: treatment introduction at week 12)

^a^ For women of childbearing potential, until 3 months after study close-up

^b^ Drug dispensation: 3 bottles for 8 weeks (8w) or 2 bottles for 4 weeks (4w)

All patients will undergo a visit (physical, “on-the-spot” visit or via secured phoning software or phone calls, according to the step-specific participant timeline), every 4 weeks until W24, when the intervention will be stopped. Assessments before starting the intervention will be compared to those after the introduction of the intervention.

The individually randomized stepped-wedge trial design is increasingly being used in clinical practice, often in rare diseases for which patient recruitment may be difficult. Because of this very rare disease, randomizing the first topical sirolimus-treated LMLM patients seemed important to obtain high-quality evidence.

Other advantages of the stepped-wedge approach include the following [34, 35]:

- Each patient is his own control, hence increasing trial power with a small patient count, while taking into account a possible persisting treatment effect (contrary to cross-over designs)
- Being able to evaluate the effect of the time of treatment introduction and treatment duration owing to the varying treatment courses from 12 to 24 weeks.
- Increasing trial acceptability in this rare disease. Indeed, during the study, participants need to comply with a significantly tighter clinical follow-up than during usual care and might be reluctant to apply for trial inclusion if they believe they will not receive the experimental treatment, especially if they live far from the study center. Resorting to phone calls instead of traditional face-to-face visits also aims to address this matter.

We could not use a placebo drug because of pharmaceutical stability issues. As a result, neither the patient nor the investigator is blinded to the treatment introduction timepoint. However, the main outcome will be assessed on photographs by an independent blinded adjudication committee.

### Methods: Participants, interventions, and outcomes

#### Study setting

The study will involve 3 French hospital centers (University Hospital of Tours, Regional Hospital of Orléans and hospital Necker-Enfants maladies, Paris) that are currently involved in the treatment of vascular anomalies.

#### Eligibility criteria

##### Inclusion

Eligible patients will be at least 5 years old. They must have a diagnosis of LMLM of any stage (Wiegand 2009) [6], assessed by clinical examination, with or without an underlying syndromic malformation (e.g., Proteus or CLAPO syndrome). Deep infiltration must have been assessed by head-and-neck MRI before study enrolment.

We chose a 5-year age threshold because although LMLMs are often diagnosed before age 2 years, they might not be clinically relevant in very young children, given their natural history of progressive worsening. Also, the topical administration that requires not swallowing the oral solution might be difficult in younger children with smaller oral cavities. Finally, in this study, we can use validated quality-of-life assessment instruments (Children’s Dermatologic Life Quality Index [cDLQI]), which cannot be used for children under age 4 years [36].

##### Exclusion

Patients with any of the following conditions are not included in the study:

- Lymphatic malformation that requires a continued background therapy (involving deep organs)
- Secondary lymphatic malformations (e.g., radiotherapy-induced lymphangiectasia)
- Immunosuppression (immunosuppressive disease or immunosuppressive treatment)
- Ongoing neoplasia
- Active chronic infectious disease (e.g., hepatitis B and C virus, HIV)
- Local necrosis
- Local fungal, viral (herpes simplex virus, varicella-zoster virus, etc.) or bacterial infection on the site of the LMLM (based on clinical examination)
- Previous treatment with systemic or topical mTOR inhibitors within 6 months before inclusion (oral sirolimus half-life is 60 h in adults according to Summary of Product Characteristics for Rapamune®).
- Previous treatment with oral or topical steroids within 10 days before inclusion (half-life of corticosteroids is 12–36 h)
- Known allergy to one of the components of the sirolimus solution
- Soybean or peanut allergy
- Pregnant or breastfeeding
- Female patients of childbearing potential not using a reliable contraceptive method
- Concurrent involvement in another therapeutic trial

#### Intervention

During the observation (control) period, patients will receive usual care for their LMLM, which is no treatment at this stage of the condition.

Then, after crossing over to the experimental period, the intervention is the 1-mg/mL sirolimus oral solution (Rapamune®, Pfizer), administered once daily. If the LMLM covers below an estimated <4 cm^2^ of target area, the treatment dose is 0.5 mL, once daily. If the LMLM covers above an estimated ≥4 cm^2^ of target area, the treatment dose is 1 mL, once daily.

A total of 0.5 or 1 mL non-diluted sirolimus solution will be taken from the bottle by using the 1-use dosing syringe and applied on a standardized disposable applicator device. Then the applicator device will be gently applied, only on the LMLM, until the solution is no longer oozing from the applicator device, for at least 10 s. The patient must then not eat, drink, brush teeth, or use mouthwash for 1 h.

Applicators and syringes are to be disposed of adequately. Rapamune® bottles must be brought at every dispensation visit to assess patient observance. Syringes must not be re-used.

The treatment will be started at a randomized timepoint (W0, W4, W8, or W12) and will be taken until W24 whatever the starting time.

In case of serious adverse reactions, including local necrosis or any systemic reaction, the intervention (topical sirolimus) will be stopped. In case of local side effects, the intervention might be temporarily halted. If the patient, the sponsor, and the investigator deem it appropriate, the intervention can be resumed, at full or half-dose. In case of relapse, the lowest bearable dose will be sought; otherwise, the treatment might be stopped. If the lesion is totally removed before W24, the investigator could propose early withdrawal of the treatment. In this setting, follow-up would be according to the protocol until W24.

Participants (or together with their parents if they are < 18 years old) will be asked to keep a daily participant diary, recording information about adherence and safety data. They will be asked to bring the diary at each study visit.

At each on-site study visit, participants will be asked to provide all their used and unused study drug containers.

If a participant were to prematurely discontinue the intervention, whatever the reason, or if adherence to intervention schedule were to be imperfect, all assessments planned should be performed, including distant and on-site monthly visits and blood tests.

Regarding concomitant care, in the event of an acute flare or local or generalized infection, antibiotics may be prescribed. Systemic steroid therapy may also be prescribed along with antibiotics, for up to 3 days. However, topical immunosuppressive drugs or steroids applied on the target area, interventional procedures (sclerotherapy, laser, radiofrequency) or surgery of the target area, and systemic steroids for more than 3 days, or immunosuppressive therapy, including systemic mTOR inhibitors, are prohibited during the study. If any of the above is administered, then the investigator must be informed, and the intervention must be stopped. In any case, all assessments planned should be performed.

#### Outcomes

##### Primary outcome

The primary outcome will be the evaluation of global severity of the LMLM by 3 independent experts (i.e., adjudication committee). To this end, the experts will compare the LMLM by using a 6-point physician’s global assessment (PGA) scale. The PGA score ranges from 0 (clear) to 5 (severe).

**Fig. 1:**
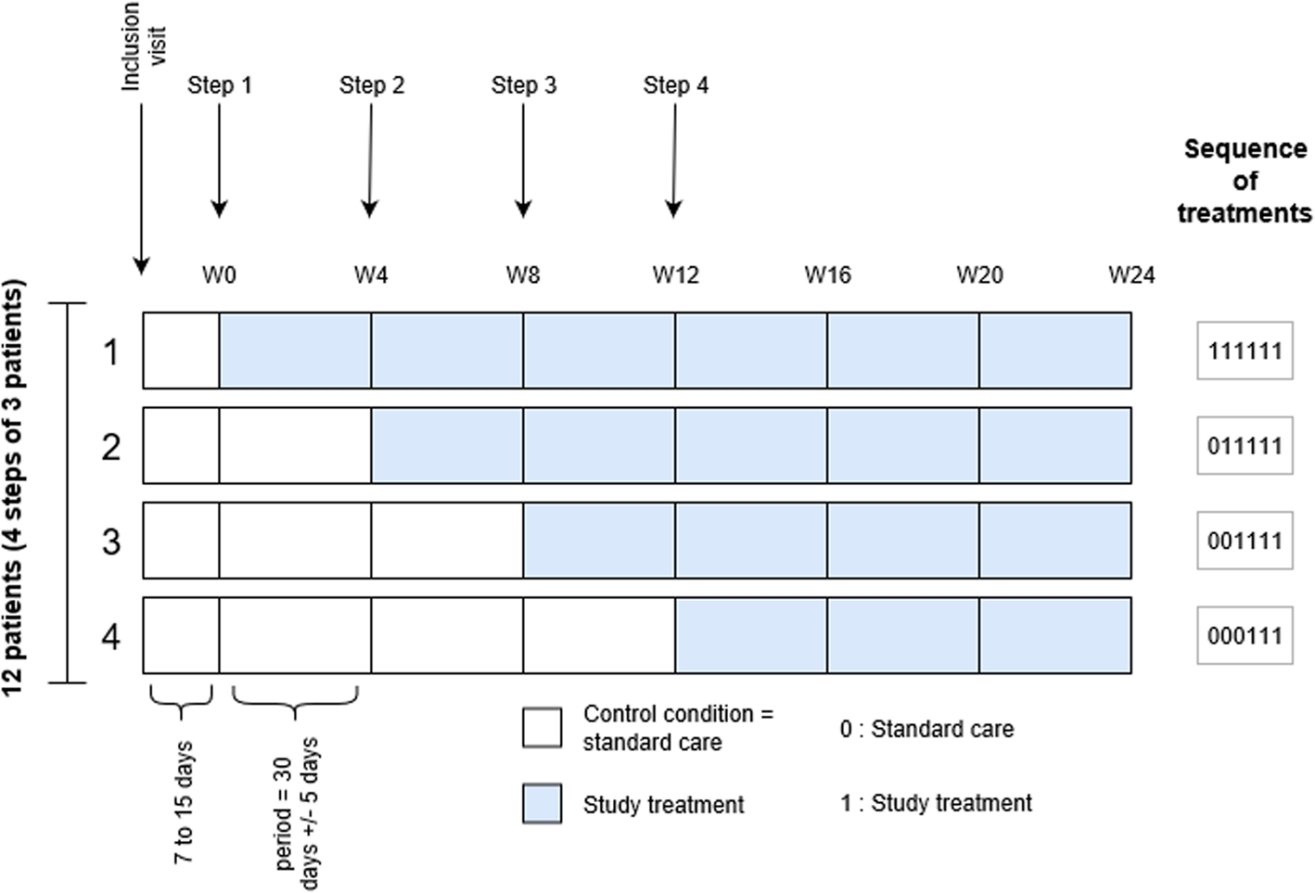
Study diagram

Photographs will be taken at baseline and every 4 weeks up to W24 by using a standardized photograph protocol (3 photos: 2 front photos [distances 8–10 and 25–27 cm] and 1 side photo [distance 8–10 cm]). Patients (and their families if relevant) will be trained in the photograph protocol during the inclusion visit and will be given cardboard templates displaying distances and angles required for each photographing incidence. During on-the-spot visits in the study center, photographs will be taken by a trained research nurse or investigator. When at home, photographs will be taken by the patient themselves or their family members and sent to a secured email address. The quality of the photographs will be controlled immediately by the investigator, and the photograph protocol might be resumed until the quality is deemed satisfactory. Then, the photographs will be immediately anonymized (Fig. 1).

The adjudication committee will be blinded to treatment allocation. They will quantify disease severity for each photograph by using the PGA scale.

There are no specific scores for LMLMs. The only existing score for head-and-neck lymphatic malformations is the Cologne Disease Score [8], but it focuses on disease-induced morbidity, with no morphology-related item, and some of its items (feeding impairment, respiration impairment) may apply to more severe lesions we do not aim to investigate. However, the PGA score is an easy-to-understand and widely used instrument in dermatology [37, 38]. We believe it relevant for this condition. The PGA score ranges from 0 (clear) to 5 (severe). A 1-point improvement versus baseline in PGA scale would already be of clinical relevance.

##### Secondary outcomes

Secondary outcomes will assess topical sirolimus efficacy and safety. They include investigator- and patient-reported outcomes, with an emphasis on quality of life and symptoms.

- Investigator-assessed PGA at weeks 0, 4, 8, 12, 16, 20, and 24
- Patient-assessed oozing, bleeding, sialorrhea, eating impairment, taste modification, aesthetic impairment, pain, and global discomfort, each on a numeric scale from 0 to 10 (0, clear; 10, very severe), at weeks 0, 4, 8, 12, 16, 20, and 24
- Global evolution assessed by the patient from −10 to 10 (−10, severe worsening; 0, no change; 10, complete recovery), at weeks 4, 8, 12, 16, 20, and 24
- Quality of life assessment (cDLQI for patients 5 to 16 years or DLQI > 16 years), at baseline, treatment introduction visit and W24
- Measurement of the lesion (length, width, thickness) by the investigator, at baseline, treatment introduction visit and W24
- Time to obtain optimal results (i.e., time from treatment introduction visit to time reaching the minimal PGA score)

Reported side effects with oral sirolimus treatment include fatigue, headache, oral mucositis, hypertension, dyslipidemia, hyperglycemia, cytopenia, and infections [nadal2016]. Topical application of sirolimus in oral diseases has also been tied to local irritation and pain [Soria2009]. Hence, to account for these reported adverse effects, safety assessments will be both clinical and biological. The clinical surveillance will include the recording of clinical local and general side effects according to the daily completion of the patient follow-up diary, questions from the investigator at each visit and physical examination and blood pressure measurement at each on-the-spot visit. Participants will also be weighed at each on-the-spot visit.

Residual blood sirolimus concentration will be monitored at week 4, week 8, and every subsequent 8th week following the beginning of the intervention (see Tables 1, 2, 3 and 4). Other routine biological safety assessments will be performed at W8, W16, and W24 of exposure, compared to baseline. Although we do not expect an important systemic diffusion, the biological surveillance measurements will mimic those used in usual systemic sirolimus treatment monitoring: complete blood count, liver (ASAT, ALAT, GGT) and renal (serum creatinine) function, lipids [triglycerides and cholesterol (i.e., total cholesterol, high-density lipoprotein cholesterol and low-density lipoprotein cholesterol estimated according to Friedewald’s formula)], and glycaemia.

Blood tests will only be used for the biological surveillance. No genetic or molecular analysis will be performed, and no specific sample conservation procedures have been designed.

#### Participant timeline

The duration of participation is 24 weeks for each patient, whatever the step they are randomized in. The participant timeline encompasses general and step-specific schedules, which are described in Tables 1, 2, 3 and 4. Even if the intervention is stopped early, whatever the reason, any assessment planned should be performed, including blood draws, phone calls and study center visits.

#### Sample size

We could not define a reasonable effect-size hypothesis because LMLM is a very rare disease, with currently no described pharmaceutical treatment for LMLM. Hence, we did not perform any sample size calculation.

#### Recruitment

Both children > 5 years old and adults are recruited alike in TOPGUN because LMLMs can be found at every age. The University hospital of Tours is a tertiary reference center for cutaneous vascular anomaly care, which all investigators are involved in. We also reached out to other university hospital centers in the “Grand-Ouest” region in France and the Necker-Enfants Malades pediatric hospital, located in Paris (authorization granted in January 2021).

### Methods: Assignment of interventions

#### Allocation

##### Sequence generation and allocation concealment mechanism

Time of cross-over to the intervention will be randomly assigned to the patient at W0, W4, W8, or W12 with a 1:1:1:1 ratio allocation as per a computer (SAS-based)-generated randomization schedule. Participants will be randomized by using Ennov Clinical, an online central randomization procedure. To ensure allocation concealment, randomization will not be possible until the participant has been recruited into the trial, especially with all selection criteria collected and met.

##### Implementation

The allocation sequence will be generated by a statistician not involved in the recruitment or follow-up of participants.

#### Blinding

We could not use a placebo drug because of pharmaceutical stability issues. Sirolimus oral solution is stable for < 30 days after opening. A recondition in unlabeled study bottles, which would be the *sine qua non* condition to consider a placebo drug, would have required very frequent treatment deliveries, which did not fit with our monthly stepped-wedge design and would have sharply increased study costs. As a result, neither the investigators nor participants will be blinded and there will be no placebo intervention. Both the patient and the investigator will be aware of his/her allocated time of cross-over to intervention. However, the adjudication committee, which will assess the primary outcome, will be blinded to treatment allocation by the use of standardized anonymized photographs.

### Methods: Data collection, management, and analysis

#### Data collection methods

LMLMs are a rare skin disease for which neither evaluation guidelines nor purposely developed evaluation instruments exist. The PGA scale is a tool widely used in dermatology. It represents a global evaluation of disease severity. We use a 6-point PGA scale: 0, clear; 1, almost clear; 2, mild; 3, moderate; 4, severe; 5, very severe. The PGA scale is widely used in psoriasis but is also used in a wide array of skin diseases [37, 38] and as an easily obtainable global evaluation of disease severity is an interesting tool. The DLQI and cDLQI are widely used instruments to assess health-related quality of life in dermatology. The cDLQI is validated for children from age 4 years up to 16 years [35].

Study staff with their own access right to the study database will enter/capture data from source documents corresponding to a participant into the protocol-specific electronic Case Report Form (eCRF). All information required by the protocol will be entered in the eCRF, and an explanation will be provided for each missing piece of information. The data must be collected as they are obtained and transcribed into these forms in a clear manner. If a correction is required for an eCRF, the time and date stamps track the person entering or updating eCRF data and create an electronic audit trail.

The participant will be given detailed contact information of their study center. In case of tolerance issues or difficulty to perform intervention procedures or self-reported outcomes collection, participants will be instructed to reach out to the study center. Any information needed will be provided, and additional unplanned visits may be organized if deemed necessary.

In case of loss of follow-up or missing data, study personnel may reach out to the participant to try to recover missing information or replan missed eventual study visits.

#### Data management

Data management will be performed by the INSERM CIC-P 1415. An eCRF will be developed by using the Ennov Clinical software. eCRF management will be performed in agreement with the INSERM CIC-P 1415 Standardized Operating Procedures (SOP). The Clinical Research Associate in charge of the study will be trained in the eCRF and will be in charge of the investigator training. Data will be entered in investigating centers via a secure web site, monitored by Clinical Research Associates and potential queries will be edited by data managers, in agreement with a prespecified data management plan.

Data will be reviewed before the database is locked. The database will be locked in agreement with the INSERM CIC-P 1415 SOPs, and data will be extracted in a SAS or other format according to statistical requirements.

#### Statistical methods

A detailed analysis plan will be a priori defined. SAS 9.4 and R 3.3.1 (or latest versions) will be used for analysis. The level of statistical significance will be set at 5%.

The primary outcome (PGA) will be treated as a continuous variable. For analysis, we will use the model described by Hooper et al. [35] Analysis will rely on a mixed linear regression model with a random effect for participant. For each participant, we will include the 7 PGA measurements from W0 to W24 (assessed every 4 weeks).

The model will be as follows, denoting *Y*_*ijt*_ as the PGA for individual *i* = 1,.., n in sequence *j* = 1,…, T at time *t* = 0,…, *T* after randomization:

$${Y}_{ij t}=\beta +{\tau}_t+{A}_{jt}+{\eta}_{ij}+{\varepsilon}_{ij t}$$

where ${\eta}_{ij}\sim N\left(0,{\sigma}_b^2\right)$ is the participant random effect; ${\varepsilon}_{ijt}\sim N\ \left(0,{\sigma}_w^2\right)$ is the random error; and *η*_*ij*_ and *ε*_*ijt*_ are all independent, and,

$${A}_{jt}\left\{\begin{array}{c}0\ if\ sequence\ j\ is\ in\ the\ control\ condition\ at\ time\ t\\ {}{\alpha}_k if\ sequence\ j\ crosses\ to\ the\ intervention\ between\ times\ t-k\ and\ t-k+1,k\ge 1\end{array}\right.$$

The mean of PGA for an individual participant follows a trajectory with baseline *β* (mean PGA at baseline) and time effects *τ*_*t*_, *t* = 0,…,6 with *τ*_0_ = 0.

When the participant crosses over to topical sirolimus, additional effects of treatment will be estimated with *α*_1_ additional treatment effect in the first 4-week period after crossing over, changing to *α*_2_, *α*_3, …,_ *α*_6_, mean additional treatment effect at W8, W12, W16, W20, and W24 after the introduction of sirolimus respectively.

This trial has a 4-sequence design, and our primary treatment effect of interest will be α_3_, the mean difference in PGA at 12 weeks after the introduction of topical sirolimus. We will also estimate *α*_1_, *α*_2_, *α*_4_, *α*_5_, and *α*_6_, mean additional treatment effects at W4, W8, W16, W20, and W24, respectively, after the introduction of sirolimus.

Missing data will not be imputed for this analysis.

Individual trajectories of PGA will be plotted by using a spaghetti plot.

Secondary outcomes (global severity of disease and functional impairments) will be treated as continuous variables ranging from 0 to 10. We will use the same model as for the primary outcome.

Time to optimal results will be described with median and interquartile range. Safety data will be reported using descriptive analysis.

### Methods: Monitoring

#### Data monitoring

A clinical research technician will be responsible for logistics of the study, producing reports concerning its state of progress, ensuring eCRF completion and update (request for additional information, corrections, etc.), and reporting severe adverse events to the sponsor. The person will work in accordance with the SOP.

A Data Safety Monitoring Board (DSMB) will consist of 2 dermatologists and 1 pharmacologist. The DSMB is an advisory committee that discusses the benefit/risk ratio of the study and the implementation of a clinical trial with the sponsor and the coordinating investigator of the study. The board will be systematically questioned, at any time by the sponsor for each case of suspected unexpected serious adverse reaction (SUSAR), once a year, before sending the safety report to French Health Authorities and if data may change the benefit and risk ratio during the clinical trial.

At any time, the sponsor may refer to the DSMB to adjudicate whether an event is an SUSAR or a severe adverse event when it is difficult to analyze or if new data change the benefit and risk ratio during the clinical trial. The DSMB will analyze the transmitted data and may request additional information and will make recommendations about the future of the clinical trial (continuation, amendments, termination).

A clinical research associate appointed by the sponsor will regularly visit each study center according to the monitoring plan depending on the frequency of inclusions and at the end of the study. During these visits, informed consent, compliance with the study protocol, and quality of the data collected in the eCRF will be reviewed.

#### Harms

All serious and non-serious events will be reported on the adverse event reporting form (initial or follow-up declaration), as thoroughly as possible, within the regulatory time limits for reporting. All adverse events will be monitored until they are completely resolved. The investigator will immediately notify the sponsor of any serious adverse event. SUSARs will be reported to Eudravigilance (European pharmacovigilance database), the French Health Authorities (ANSM), and the investigators.

#### Auditing

An audit may be performed at any time by people appointed by the sponsor who are independent of those responsible for the study. The audit aims to ensure the good quality of the study and that the law and regulations in force are being observed. The investigators agree to comply with the requirements of the sponsor and the relevant authority for an audit or an inspection of the study.

The audit can apply to all stages of the study, from development of the protocol to publication of the results and filing the data used or produced in the study.

### Ethics and dissemination

#### Research ethics approval

The sponsor and the investigator or investigators undertake to conduct this study in compliance with French law in force (*Code de Santé Publique*), the recommendations of French and international Good Clinical Practices, the Helsinki Declaration (Ethical Principles for Medical Research involving Human Subjects), and the European regulations related to clinical research. The study will be conducted in accordance with this protocol. With the exclusion of emergency situations requiring specific therapeutic actions, the investigators undertake to observe the protocol in all respects.

This research is registered in the European EudraCT database in accordance with article L1121.15 of the French Public Health Act.

This protocol has been granted both ANSM (French Regulatory Authority) and CPP (*Comité de Protection des Personnes*, French Ethical Review Committee) approval (19.03.28.46025).

Copy of the ethics committee agreement is found in Additional file 1.

#### Protocol amendments

Important protocol modifications will be submitted for approval to the institutional review board of the University Hospital of Tours and will be communicated to coinvestigators.

The protocol was amended on December 12, 2019. Inclusion criteria were modified to LMLM of any stage with lingual involvement, provided sirolimus is only applied on the anterior part of the tongue (before the vallate papillae). The exclusion criterion “previous use of systemic sirolimus in the last 12 months” was modified to “previous use of systemic sirolimus in the last 6 months,” which is still well above a 7 half-life (i.e., 420 h = 3 weeks) clearance threshold. The intervention was not modified. The aim of the amendment was to make screening easier in this very rare disease.

A second amendment has been authorized on January 22, 2021. It adds the Necker-Enfants-Malades Hospital as a study center and extends the recruitment period by 1 year.

#### Consent or assent

During a routine pre-screening visit, eligible subjects will be orally informed of the aim of the protocol and its procedures. Age-specific information letters will also be provided to the subject (and their legal tutors if applicable) by the investigator and a reflection period will be respected before consent collection.

Informed consent will be obtained from the participant and, if applicable, parents or legal tutors, by the investigator, during the first visit (p27, see Tables 1, 2, 3 and 4), before any assessment or protocol procedure. The investigator will be a physician, either a dermatologist or a facial surgeon.

The written and informed consent of the patient, if obtained, must be dated and signed both by the patient (or their parent or legal representatives) and the investigator before any further study intervention. Children ≥ 16 years old must also consent to use of their data. The patient will receive a copy of the signed written consent and information letter. The original Information Letter and Consent Form will be kept by the investigator (even if the patient moves to a new hospital during the study) in a safe place inaccessible to third parties. The consent form will be signed before any intervention needed for the study.

#### Confidentiality

In accordance with the legislative provisions in force (articles L.1121-3 and R.5121-13 of the French Public Health Code), people with direct access to source data will take all necessary precautions to ensure the confidentiality of information relating to the study intervention, research studies, and people taking part in them, particularly in regard to their identity and the results obtained. During the study or when it is over, the information collected on the people taking part in it and forwarded to the sponsor by the investigators (or any other specialized staff member involved) will be made anonymous. Under no circumstances must the uncoded names or addresses of the people concerned appear in it.

#### Access to data

The investigator will prepare and maintain adequate and accurate source documents designed to record all observations and other pertinent data for each participant of the study. The sponsor is responsible for obtaining the agreement of all the parties involved in the study in order to guarantee direct access in all the sites where the study is being conducted to source data, source documents, and reports, so that he/she can control their quality and audit them.

#### Dissemination policy

Any written or oral communication of the results of the study will be previously agreed by the coordinating investigator and, if necessary, by the scientific committee constituted for the study. Publication of the main results will mention the sponsor and the funding source. We will follow the Recommendations for the Conduct, Reporting, Editing, and Publication of Scholarly Work in Medical Journals (updated in December 2015) from the International Committee of Medical Journal Editors. All investigators who are not cited in the authorship will be listed as non-author contributors. In accordance with the law no. 2002-303 of March 4, 2002, participants will be informed, at their request, of the overall results of the study.

#### SPIRIT statement

This protocol has been written in accordance with the Standard Protocol Items: Recommendations for Interventional Trials (SPIRIT) guidelines [39]. The SPIRIT checklist is found in Additional file 2.

## Discussion

This trial is the first and currently sole ongoing trial to our knowledge to investigate topical sirolimus in mucosal LM, namely LMLMs. LMLMs are a rare disease, and patients often carry a heavy symptomatic burden, even with smaller-sized malformations. In particular, even with mild superficial lesions, the aesthetic component might be of significant importance for young patients of school age.

There are currently no other therapeutic options for LMLMs than surgery or interventional procedures such as laser or radiofrequency ablation, which are all painful procedures that carry the risk of performing general anesthesia in children and often yield incomplete results with a significant rate of local relapses. Moreover, surgery may be debilitating in the oral cavity in children, and laser or radiofrequency ablation, although showing temporary efficacy, may hinder further chances of complete surgical excision by inducing deep-tissue fibrosis [6].

Thus, there is an unmet need for an easily carriable medical treatment with few side effects. Such a treatment could be used as a first-intent monotherapy for smaller LMLMs or before a further planned surgical excision in the watchful-waiting approach to more severe LMs.

Sirolimus has already shown efficacy as a systemic therapy for LM and carries interesting promise as a cutaneous topical treatment. Despite its high molecular weight, 914.17 Da, which may hinder transcutaneous diffusion, relevant clinical results with varying formulations could be achieved, with concentrations ranging from 0.015 to 8% [29, 40]. From previous pharmacokinetic studies [33] and case series in oral inflammatory diseases [25, 26], we believe that therapeutic-range local concentrations can be obtained with daily application of topical sirolimus on the tongue, with clinical improvement as a result, although such an intervention has yet to be reported in LM.

Given both the rarity of LMLM and the novelty of topical sirolimus, we planned the TOPGUN trial to assess the safety and efficacy of topical sirolimus in LMLMs.

Despite the low recruitment capacity, we wanted this trial to be able to produce good-quality, randomized evidence [41]. The individually randomized stepped-wedge design allows for using the patient as their own control and to perform repeated outcome measurements to boost statistical power, even in the absence of data regarding a carry-over effect after treatment discontinuation.

Also, we ensured a significant emphasis on trial acceptability. Patients under watchful waiting in our tertiary center usually undergo a once- or twice-a-year follow-up, and they may come from several dozens or hundreds of kilometers away. Hence, we designed a trial that minimizes on-the-spot study center visits while maintaining both the required close clinical and biological surveillance and outcome assessor blinding (by resorting to an external adjudication committee). Similarly, the stepped-wedge design allows every patient to benefit from the experimental treatment regimen.

Indeed, although safety data in the literature is reassuring, notably the very low sirolimus systemic passage, data on the exact setting of topical application in LMLMs are lacking. Safety procedures were then designed for a “worst-case scenario” of a total sirolimus systemic diffusion, which would amount to an oral 0.5- to 1-mg per day oral sirolimus regimen. We also gathered a DSMB in that respect.

In the end, if the TOPGUN trial demonstrates a relevant clinical benefit and a good safety record, it will be supported by good-quality evidence and could pave the way for a rapid change in firstline clinical practices in LMLMs.

### Trial status

The current version of the protocol is v3.0, dated 8 December 2020. The first inclusion occurred on 14 January 2020, with a due recruitment period of 60 months (i.e., ends 14 January 2025).

Five patients have been included, with two of them having completed the study.

## Supplementary Information

**Additional file 1.** Copy of the ethics committee agreement.

**Additional file 2.** The SPIRIT checklist.

**Additional file 3.** Copy of the original funding document.

## Data Availability

Deidentified individual participant data (including data dictionaries) will be made available, in addition to study protocols, the statistical analysis plan, and the informed consent form: the data will be made available upon publication to researchers who provide a methodologically sound proposal for use in achieving the goals of the approved proposal. A written agreement must be signed with the University Hospital of Tours and the coordinator of the study. Proposals should be submitted to dpo@chu-tours.fr.

## Abbreviations

AKT: Protein kinase B
ALAT: Alanine aminotransferase
ANSM: Agence Nationale de la Sûreté du Médicament *(French national drug safety agency)*
ASAT: Aspartate aminotransferase
CIC-P: Centre d’Investigation Clinique – Polyvalent *(Clinical Investigation Center)*
CPP: Comité de Protection des Personnes *(Ethics Committee)*
cDLQI: Children’s Dermatologic Life Quality Index
DLQI: Dermatologic Life Quality Index
DSMB: Data Safety Monitoring Board
eCRF: Electronic Case Report Form
FDA: Food and Drugs Administration
GGT: γ-Glutamyl Transferase
INSERM: Institut National de la Santé et de la Recherche Médicale *(French State-funded Biomedical Research Agency)*
ISSVA: International Society for the Study of Vascular Anomalies
LM: Lymphatic malformation
LMLM: Lingual microcystic lymphatic malformation
mTOR: Mammalian target of rapamycin
PI3k: Phosphoinositide 3-kinase
PGA: Physician’s Global Assessment
SOP: Standardized Operating Procedures
SUSAR: Suspected unexpected serious adverse reaction
VEGF(R): Vascular endothelial growth factor (receptor)
