# Supplementary material for: Topical sirolimus solution for lingual microcystic lymphatic malformations in children and adults (TOPGUN): study protocol for a multicenter, randomized, assessor-blinded, controlled, stepped-wedge clinical trial
Source: Trials. 2022 Jul 8;23:557. doi: 10.1186/s13063-022-06365-y (PMC9270761; doi:10.1186/s13063-022-06365-y)
Supplement: Supplementary file 1 — Additional file 1. Copy of the ethics committee agreement. [file 13063_2022_6365_MOESM1_ESM.pdf]

# NORTH WEST PERSON PROTECTION COMMITTEE III

**CHU - level 03 - door 03-363**
**14033 Caen cedex 9**

**Tel : 09.64.08.19.44.**
**Fax : 02.31.47.57.81.**
**E-mail : cppnordouest3@orange.fr**

Caen, September 18, 2019

CHRU (Research Department)
for the attention of Karine FEVRE
75475 Paris

Ref. CPP: 2019-41
EudraCT number: 2019-001530-33
Ref. promoter: TOPGUN
Ref. CNRIPH: 19.03.28.46025
Category 1 MED

Mrs,

The NORD WEST III CPP, by deliberation dated June 29, 2019, after having taken note of the conclusions of the pediatric expert, issued a conditional favorable opinion concerning the above-referenced protocol, entitled "TOPGUN study: Evaluation of the efficacy and tolerance of sirolimus in local application on microcystic lymphatic malformations of the tongue. Pilot study in adults and children "and coordinated by Dr. Annabel MARUANI [Tours].

Seated together on June 29, 2019, under the chairmanship of Ms. Charlotte GOURIO,

<u>Members of the first college</u>
under 1 ° of article R.1123-4 of the CSP

- Mr. Rémy MORELLO, qualified person because of his competence in biostatistics or epidemiology, full member,
- Mr. Jean-François HERON, doctor, full member,
- Mr. Claude BAZIN, doctor, full member,

under 3 ° of article R.1123-4 of the CSP
- Ms. Charlotte GOURIO, hospital pharmacist, Chairman of the committee, full member,
- Ms. Valérie AUCLAIR, hospital pharmacist, substitute member,

<u>Members of the second college</u>
under Title 1 of Article R .1123-4 of the CSP

- Mr. Hervé PLATEL, person qualified in the ethical field, full member, under title 3 ° of article R .1123-4 of the CSP
- Ms. Sylvie BALP, technical advisor to the deputy general directorate of solidarity of the General Council of Caen, full member,

under title 4 ° of article R .1123-4 of the CSP
- Ms. Fanny ROGUE, lecturer at the Faculty of Law of Caen, vice-president of the committee, full member,

under Title 5 of Article R .1123-4 of the CSP
- Mr. Alain INGOUF, representative of an approved association of patients or users of the health system, alternate member,

# NORTH WEST PERSON PROTECTION COMMITTEE III

**CHU - level 03 - door 03-363**
**14033 Caen cedex 9**

Deliberation

Considering the following documents:

- response letter dated July 26, 2019 and signed,
- protocol, English version 1.2 of July 26, 2019,
- summary, French version 1.2 of July 26, 2019,
- information document (parents of minor children), version 1.1 of July 26, 2019,
- information document (children aged 5 to 12), version 1.1 of July 26, 2019,
- information document (adolescents aged 13 to 17), version 1.1 of July 26, 2019,
- information document (adolescents who have reached the age of majority), version 1.1 of July 26, 2019,
- information document (adult patients), version 1.1 of July 26, 2019,
- consent form (parents of minor children), version 1.1 of July 26, 2019,
- consent form (adolescents who have reached the age of majority), version 1.1 of July 26, 2019,
- consent form (adult patients), version 1.1 of July 26, 2019,
- ANSM authorization dated May 24, 2019,
- shooting protocol, version 01,
- patient logbook, version 01,
- participation card, version 01,

the CPP NORD OUEST III therefore opted for:

| DEFINITIVE FAVORABLE OPINION |
|---|

Please accept, Madam, my best regards

Mme Charlotte GOURIO
Presidente
