# Supplementary material for: Topical sirolimus solution for lingual microcystic lymphatic malformations in children and adults (TOPGUN): study protocol for a multicenter, randomized, assessor-blinded, controlled, stepped-wedge clinical trial
Source: Trials. 2022 Jul 8;23:557. doi: 10.1186/s13063-022-06365-y (PMC9270761; doi:10.1186/s13063-022-06365-y)
Supplement: Supplementary file 3 — Additional file 3. Copy of the original funding document. [file 13063_2022_6365_MOESM3_ESM.pdf]

Department of Medical Affairs and Research

CHRU
HÔPITAUX DE TOURS

Direction de la Recherche
Directeur
Violaine MIZZI
Directeur adjoint
Julien LE BONNIEC
Tél : 02.47.47.39.99

Partenariats et valorisation
Delphine VALIN
d.valin@chu-tours.fr
Tél. 02.34.37.89.23

Délégation à la Recherche Clinique et à l'Innovation
Président
Pr Philippe GOUPILLE
philippe.goupille@univ-tours.fr
Directeur délégué
Violaine MIZZI
v.mizzi@chu-tours.fr
Directeur adjoint
Julien LE BONNIEC
j.lebonniec@chu-tours.fr
Tél : 02.47.47.39.99

Gestion et pilotage
Attachée d'Administration Hospitalière
Tél. 02.34.37.95.33

Ingénieur qualité
Coraline GADRAS
c.gadras.@chu-tours.fr
Tél : 02.47.47.59.96

Cellule d'aide au montage des projets
Guillaume FLURY
g.flury@chu-tours.fr
Tél. 02.18.37.08.57

Promotions CHU et contrôle qualité
Karine FEVRE
k.fevre@chu-tours.fr
Tél. 02.18.37.08.34
Dr Sophie GUYETANT
sophie.guyetant@chu-tours.fr
Tél. 02.34.37.89.27

Christelle MEUNIER
c.meunier@chu-tours.fr
Tél. 02.18.37.06.19
Nathalie JUTEAU
n.juteau@chu-tours.fr
Tél. 02.47.47.46.73
Secrétariat
Laëtitia EVEN
l.even@chu-tours.fr
Tél. 02.18.37.06.51

Promotions externes
promotions.externes@chu-tours.fr
Marie GRANGER
Tél. 02.18.37.06.18
Gestionnaire
Sarah REROLLE
Tél. 02.47.47.37.99
Annie FRANCO
Tél. 02.47.47.70.07

Secrétariat
Nathalie GINESTE
n.gineste@chu-tours.fr
Tél : 02.18.37.08.01
Annie FRANCO
a.franco@chu-tours.fr
Tél : 02.47.47.70.07

Doctor Antoine Marchand
Dermatology unity
CHRU Tours-Trousseau

Tours, 2018 /07/23

Reference : JLB/GF/2018-DR293
Responsible : Guillaume FLURY
Copy to : Nathalie JUTEAU

Subject : Promotion AOI 2018

Sir,

We congratulate you on obtaining funding for your "TOPical sirolimus in linGual lymphatic malformationN-TOPGUN "project at AOI 2018.

In order to continue your efforts, the CHRU de Tours naturally promotes your study. The quality control of the protocols promoted by the CHRU de Tours is entrusted to the Clinical Research Associates of the CHRU, who on this point intervene on behalf of the promoter. Also prior to any implementation, I invite you to contact Madame JUTEAU (Tel: 02.47.47.46.73), Project Manager, to agree on the terms of support for monitoring your study.

In addition, I call you that the registration of a clinical trial on the register "Clinicaltrials" has become mandatory. It is up to you to record the study before the first inclusion by filling in the various items for this research.

Please, Sir, with the assurance of my highest consideration.

Le Directeur adjoint,
Direction des Affaires Médicales
et de la Recherche,
Secteur Recherche

Julien LE BONNIEC
